# Neuropilins: A New Target for Cancer Therapy

**DOI:** 10.3390/cancers3021899

**Published:** 2011-04-08

**Authors:** Camille Grandclement, Christophe Borg

**Affiliations:** 1 INSERM UMR 645, F-25020 Besançon, France; E-Mail: christophe.borg@efs.sante.fr; 2 University of Franche-Comté, IFR133, F-25020 Besançon, France; 3 EFS Bourgogne Franche-Comté, F-25020 Besançon, France; 4 Department of Medical Oncology, CHU Besançon, F-25000 Besançon, France

**Keywords:** neuropilins, cancer, angiogenesis, lymphangiogenesis, targeted therapies

## Abstract

Recent investigations highlighted strong similarities between neural crest migration during embryogenesis and metastatic processes. Indeed, some families of axon guidance molecules were also reported to participate in cancer invasion: plexins/semaphorins/neuropilins, ephrins/Eph receptors, netrin/DCC/UNC5. Neuropilins (NRPs) are transmembrane non tyrosine-kinase glycoproteins first identified as receptors for class-3 semaphorins. They are particularly involved in neural crest migration and axonal growth during development of the nervous system. Since many types of tumor and endothelial cells express NRP receptors, various soluble molecules were also found to interact with these receptors to modulate cancer progression. Among them, angiogenic factors belonging to the Vascular Endothelial Growth Factor (VEGF) family seem to be responsible for NRP-related angiogenesis. Because NRPs expression is often upregulated in cancer tissues and correlated with poor prognosis, NRPs expression might be considered as a prognostic factor. While NRP1 was intensively studied for many years and identified as an attractive angiogenesis target for cancer therapy, the NRP2 signaling pathway has just recently been studied. Although NRP genes share 44% homology, differences in their expression patterns, ligands specificities and signaling pathways were observed. Indeed, NRP2 may regulate tumor progression by several concurrent mechanisms, not only angiogenesis but lymphangiogenesis, epithelial-mesenchymal transition and metastasis. In view of their multiples functions in cancer promotion, NRPs fulfill all the criteria of a therapeutic target for innovative anti-tumor therapies. This review focuses on NRP-specific roles in tumor progression.

## 1. Introduction

Neuropilins (NRPs; previously known as A5 protein) were first identified by Takagi *et al.* in 1987 by immunofluorescent staining of frozen sections of *Xenopus* tadpole nervous system [1]. This glycoprotein of 130–140 kDa, highly conserved among vertebrates, was then isolated in the nervous developing system of a broad spectrum of animal species, such as chicken [2,3], mice [4], and rats [5,6]. While NRP1 was the first member of the NRP family to be described, NRP2 was rapidly isolated by Chen *et al.* in 1997, by RT-PCR and gene transfer [7].

**Figure 1.**
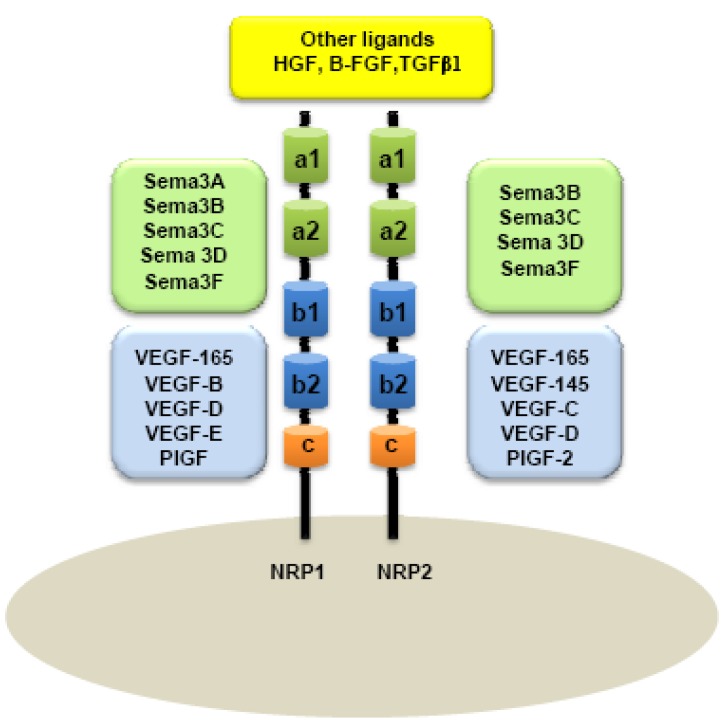
Neuropilins (NRPs) and their ligands. Class-3 semaphorins bind a1/a2 sub-units (green) whereas vascular-endothelial growth factors preferentially bind b1/b2 sub-units (blue). Other growth factors such as HGF, B-FGF, TGFβ1 have been recently reported to bind both NRPs (yellow).

A major distinction between these two members of the NRP family is based on their ligand specificities. NRPs were originally described as high-affinity cell-surface receptors for axon guidance molecules such as class-3 semaphorins (Sema) [6]. Indeed, NRP1 is a receptor for semaphorin-3A, 3C, 3F [5,6] while NRP2 preferentially binds Semaphorin 3B, 3C, 3D, 3F [7,8] (Figure 1).

Several analyses using mutant mice lacking NRPs function subsequently conferred to semaphorin/neuropilin an essential role in axon guidance during nervous system development [8-11].

*In vivo* models using NRPs transgenes also suggested other essential functions of NRPs. Indeed, overexpression of NRP1 in chimeric mice generated an excess of capillaries and blood vessels, suggesting an important role of NRP1 in angiogenesis and vasculogenesis [12]. In contrast, NRP1 null-mutant embryos showed severe types of vascular defects, especially in neuronal vasculature, yolk sac vessel network organization, aortic arch development [13] and in the cardiovascular system, resulting in death of homozygous embryos at E12.5 to E13.5 [13,14]. NRP2 knock-out mice are viable suggesting that NRP2 is not essential for vascular development, unlike NRP1 [9,11]. Moreover, NRP2 homozygous mutant mice are characterized by abnormal lymphatic and capillary development suggesting a selective requirement for NRP2 in the formation of lymphatic vessels [15]. However, double knock-out of NRPs genes (NRP1^−/−^ NRP2^−/−^) constitutes the most severe phenotype observed, impairing any blood vessel development and causing earliest death *in utero* at E8.5 [14].

Because Vascular Endothelial Growth Factor (VEGF) plays a central role in the development of vascular network, interactions between NRPs and VEGF were rapidly considered. NRPs were indeed found to be receptors for several members of the VEGF family. NRP1 can effectively bind VEGF_165_, PIGF-2 (Placenta Growth Factor), VEGF-B, VEGF-C, VEGF-D and VEGF-E [16-21], whereas NRP2 is a receptor for VEGF_145_, VEGF_165_, PIGF-2 [18,22], VEGF-C [20,22], and VEGF-D [20]. NRPs are also reported to bind diverse heparin-growth factors, such as FGF (Fibroblast Growth Factor) and HGF (Hepatocyte Growth Factor) [23,24] (Figure 1).

## 2. NRPs: Structural Particularities

In humans, NRP1 and NRP2 genes map to two different chromosomes: Chromosomes 10p12 and 2q34, respectively [25]. Although NRPs share only 44% homology in their amino acid sequences, some similarities to known proteins can be observed in their structure. NRPs are composed of an extracellular domain, transmembrane domain and a short intracellular domain. Indeed, the extracellular domain is composed of two Complement Binding motifs (CUB), homologous to the C1r and C1s complement components (named domains a1 and a2), two domains b1 and b2 homologous to the coagulation factors V and VIII and one third domain, c, homologous to the meprim domain sharing a tyrosine phosphatase activity μ [4,26]. a1/a2 domains are responsible for semaphorin binding, whereas b1/b2 are suggested for both VEGF and semaphorin binding. c-domain is involved in dimerization of the receptor [8] (Figure 1). Because NRPs have a short intracellular domain of only 40 amino acids, it was assumed that they cannot transmit any signal on their own.

### 2.1. Isoforms

**Figure 2.**
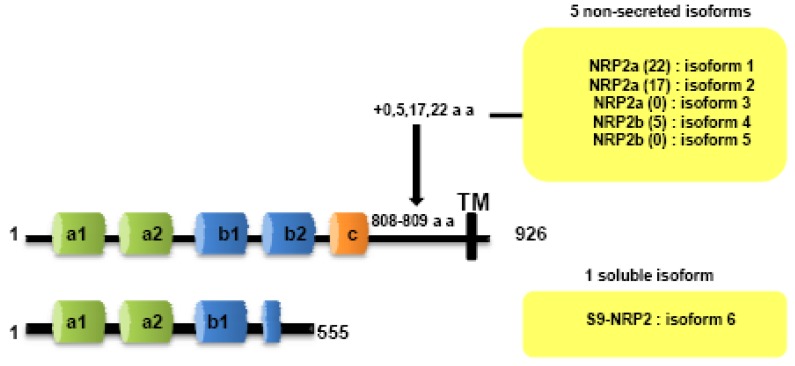
NRP2 transcript variants encode distinct isoforms.

Both NRPs genes are composed of 17 exons. Contrary to NRP1, NRP2 is expressed as several alternatively spliced forms. In particularly, two isoforms of NRP2, NRP2a and NRP2b, that arise by alternative splicing, have been described subsequently in mouse [7] and humans [25]. Divergences between NRP2a and NRP2b are principally observed in the linker between transmembrane and cytoplasmic domains. NRP2 subisoforms were subsequently described by Chen [7] and Rossignol [25]. Insertions of 17 or 22 amino acids after amino acid 809 are described for NRP2a (NRP2a _(17)_, NRP2a_(22)_) whereas NRP2b is characterized by insertions of 0 or 5 amino acids after amino acid 808 (NRP2b_(0)_, NRP2b_(5)_) (Figure 2). NRP2a seems to be closer to NRP1 (44% homology) than NRP2b (11%) [25].

### 2.2. Soluble Forms

Two soluble forms of NRP1 (s_11_NRP1 and s_12_NRP1) and one of NRP2 (_s9_NRP2) were cloned by Rossignol and collaborators [25]. Later, two novel soluble forms of NRP1, _sIII_NRP1 and _sIV_NRPI were characterized [27]. While these soluble isoforms have conserved their extracellular domains responsible for ligand binding, c-domain, transmembrane and intracellular domains were lacking. Moreover, Gagnon *et al.*, reported that s_11_NRP1 is capable of tumor cell apoptosis by antagonizing VEGF binding, suggesting that sNRPs and NRPs have opposite functions [28].

## 3. Neuropilins Expression Pattern

### 3.1. Embryogenesis

First reports limited NRPs expression in the nervous developing tissues [1,2,4,7,29]. Indeed, Chen *et al.* observed increased NRP2 expression in most components of the developing nervous system including spinal cord, sympathetic ganglia, olfactory system, neocortex, hippocamp [7].

NRP were also found in development of many non neuronal tissues such as bones, several muscles, intestinal epithelium, kidney, lung, dorsal aorta [7]. Moreover, knock-out studies have suggested an important role of the NRPs in the development of the vascular system during embryogenesis. While NRP1 is preferentially expressed in arteries during embryonic development, NRP2 is required for the formation of veins and lymphatic vessels [12,15].

### 3.2. Immune System

NRP1 was rapidly identified on various immune cells such as some subpopulations of T lymphocytes and on dendritic cells (DC) *in vitro* and *in vivo* [30]. In this immune context, NRP1 enhances cell-cell interaction, especially in mediating DC-induced proliferation of resting T cells [30]. NRP1 is expressed by CD4+CD25+ murine regulatory T cells but not by naive T cells [31]. When expressed on murine T reg cells, NRP1 inhibits T cell proliferation [32]. However, Milpied *et al.* observed in 2009 that NRP1 expression on murine T reg could not be extended in human [33]. On the other hand, NRP2's contribution in the immune system was only very recently studied. NRP2 is expressed on a polysialylated form on mature human DC [34]. Because polysialylation of proteins is a very rare phenomenon, its role has not been extensively characterized. However, polysialylation of NRP2 on DC seems to be essential for CCL21-dependent DC migration (CCL21: Chemokine C-C motif Ligand 21) to the lymph nodes during immune response [35,36].

### 3.3. Human Tumors

The contribution of NRPs in angiogenesis prompted the investigation of NRP's role in oncogenesis. Besides the presence of NRPs on tumor-associated vessels, authors have reported the wide expression of NRPs among different human tumors, suggesting a potential role of this molecular network in cancer progression. In 1998, Soker *et al.* isolated NRP1 from endothelial cells and tumor tissues [21]. Indeed, NRPs expression is not restricted in intra-tumoral vessels, but a large variety of cancer cells are reported to express one or both NRPs. Moreover, NRPs are often the only VEGF-receptors expressed by tumor cells [37,38], conferring an essential role of these glycoproteins as growth factor receptors. Although NRP1 is expressed by a large variety of tumors, even less is known concerning the expression of NRP2 (Table 1). However, NRP2 expression was found in osteosarcomas [39], melanomas [40], lung cancers [41,42], brain tumors [43,44] colon cancers [45], pancreatic cancers [46-49], breast cancers [50], myeloid leukemias [51], salivary adenoid cystic carcinomas (SACCs) [52], infantile hemangiomas [53], ovarian neoplasms [54] and bladder cancers [55] (Table 1).

### 3.4. Regulation of Neuropilins Expression

NRP1 expression was promoted by hypoxia in several models [78-80] and by ischemia in rats [81], and in mice [82]. Moreover, several growth factors and inflammatory cytokines are involved in NRP regulation too: In pancreatic cancer cells, IL-6 enhances NRP1 expression [60] whereas IL-8 increases NRP2 expression via activation of ERK1/2 pathway [83]. TNFα was shown to upregulate VEGFR2 and NRP1 in human vascular endothelial cells [84]. While TGF-*β*1 and IL-1*β* inhibit NRP1 expression, TGF-*β*1 stimulates NRP2 expression in human proximal tubular cells through activation of MEK1/2-ERK1/2 pathway [85]. Oncostatin M activates both NRP1 and NRP2 expression [85].

## 4. Neuropilins Role in Oncogenesis

NRPs display a short intracytoplasmic tail of 40 amino acids which does not contain any kinase domain, leading to the suggestion that neuropilins can not directly transmit intracellular signals. This led to the proposal that hetero-dimerization with other membrane receptors are required to mediate neuropilin-downstream signaling.

### 4.1. Interactions with Plexins/Semaphorins

Semaphorins (Sema, also known as collapsins) are subdivided into eight classes, on the basis of structural similarities. Class 1 and 2 constitutes invertebrate semaphorins, whereas classes 3 to 7 comprise vertebrate semaphorins [86]. All semaphorins are characterized by an identical N-terminal 500-amino-acid-long sema domain, which is essential for semaphorin signaling. The structure of the sema domain is a seven-blade β-propeller fold which presented similarities with extracellular domain of α-integrins [87]. Next to the sema domain, semaphorins contain several distinct domains in their structure, such as a plexin-semaphorin-integrin domain (PSI), an immunoglobulin-like, a thrombospondin and a basic-C domains [88]. Class-3 semaphorins are secreted semaphorins characterized by a basic-charged domain at the C-terminus. Class 4–7 semaphorins are membrane-bound semaphorins which are characterized by thrombospondin repeats (class-5 semaphorins) or glycophosphatidylinositol (GPI) anchor (class-7 semaphorins). Membrane-bound semaphorins can be cleaved into soluble forms through proteolytic degradation [89]. Two high affinity receptors have been identified for semaphorins: Plexins and Neuropilins. Various studies indicate that plexins are required for class 3 semaphorin/neuropilins signaling pathway during both embryonic development and tumorigenesis.

Plexin family is the first class of co-receptor identified. Plexins have been identified like NRPs, from immunostaining of *Xenopus* tadpoles nervous tissue [1]. While plexins play an important role in axon guidance [90] by forming complexes with NRPs [91,92], plexins have been identified on various tumor tissues, suggesting a role in tumorigenesis [93,94]. Nine members of the plexin family have been identified, subdivided into four subfamilies comprising four type-A plexins, three type-B plexins, plexin C1 and plexin D1. Plexins can tranduce intracellular signals through activation of Rho-like GTPases, such as Rnd1 for plexin A1 and Rac1 for plexin B1 [95-97]. Moreover, type B plexins contain a binding site for a PDZ domain in the C-terminal domain [98-100]. The extracellular domains of all plexins are characterized by the presence of a sema domain, and by the presence of PSI and glycine-proline (G-P)-rich motifs [86]. Membrane-bound semaphorins can directly bind to the plexins, whereas secreted semaphorins such as class-3 semaphorins required NRPs as co-receptor to mediate the signal [86].

**Figure 3.**
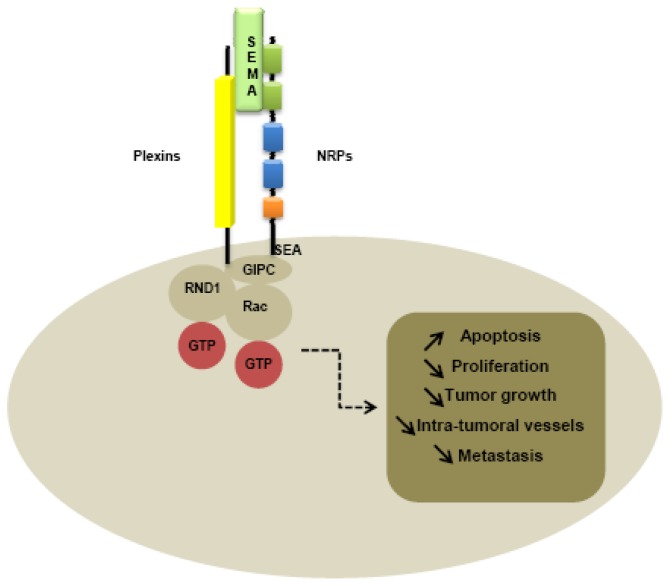
NRPs cooperate with class 3 semaphorins and plexins in endothelial and cancer cells.

Like type-B plexins, NRPs contain a binding site for PDZ domains in the C-terminal domain. Indeed, the PDZ domain of NIP, also called GIPC (GAIP interacting protein at the C terminus), is thought to be implicated in interaction with NRPs and plexins, activating small GTPase-activating proteins [101]. In particular, the last three amino-acids SEA of the C-terminal sequence of NRPs seem to be responsible for interaction with G-interacting proteins [101] (Figure 3).

Semaphorins are reported to be very often down-regulated or mutated in human cancers, allowing massive VEGF/NRPs interactions. Because semaphorins are frequently inactivated by allele loss or promoter methylation, they have been rapidly considered to function as a TSG (tumor suppressor gene). Indeed, deletions occur in the region 3p21.3 of the short arm of chromosome 3, a region encoding for Sema3B and Sema3F in various cancers, including lung cancer and even ovarian cancer [102-104]. Moreover, semaphorin promoter hypermethylation and various mutations occur in lung and breast cancers [42,105-108].

#### 4.1.1. Semaphorin 3A

First, Bagnard *et al.* reported that Semaphorin 3A (Sema3A) mediates cell repulsion and can even induce cell death in a neuroectodermal progenitor cell line, both effects depending on interactions with NRP1 [109].When Sema3A is added to the culture medium of Human Umbilical Vein Endothelial Cells (HUVEC) cells for 48 h with VEGF_165_, cell survival decreases. NRP1 is implicated in this Sema3A-mediated apoptosis [110]. Moreover, Sema3A has been implicated directly in Fas-mediated apoptosis in a recent study [111]. After a stimulation of leukemic T cells by Sema3A, Fas localizes into the lipid rafts and sensitizes these T cells to FasL-mediated apoptosis [111] (Table 2).

In another study, Kigel and colleagues transfected breast cancer cells expressing NRP1 and-or NRP2 with each semaphorin to analyze their role in tumor progression in xenograft experiments [112]. Sema3A, sema3D, sema3E and sema3G overexpression in breast cancer cells significantly inhibits the development of tumor in xenograft models and decreases the number of intra-tumor blood vessels, suggesting an anti-angiogenic role of these molecules [112]. In this model, the anti-tumor effect of each of the semaphorins correlated very well with the expression of the related receptor on tumor cells [112]. Furthermore, in a very recent study using multiple murine models of tumorigenesis, Maione and collaborators showed that inhibition of sema3A in the later stages of carcinogenesis is responsible for enhanced angiogenesis and tumor progression [113]. By contrast, restoration of Sema3A expression in these cells normalizes intra-tumor vasculature, indicating that Sema3A could be used as a potential anti-angiogenic agent [113]. In another recent study, Sema3A role in tumor progression and in tumor angiogenesis was evaluated using three experimental approaches, using different systems for the release of the semaphorins [114]. In all experiments, NRP1 seems to be essential for Sema3 A-mediated inhibition of tumor growth, angiogenesis and metastasis [114] (Table 2).

#### 4.1.2. Semaphorin 3B

In lung and ovarian cancer cells, Semaphorin 3B (Sema3B) expression decreases colony formation, proliferation, and even tumorigenicity in murine xenograft experiments [42,115]. Similarly, Sema3B was shown to induce apoptosis in cancer cells, in particularly by blocking VEGF-binding to the NRPs [116,117] (Table 2). Moreover, NRP1-Sema3B interactions induce high level of IL-8 in tumor cells, leading to a massive monocyte/macrophage recruitment, promoting invasion and metastasis formation [118]. As a consequence, when sema3B is inhibited using RNA interference and IL-8 neutralized with blocking monoclonal antibodies, a decrease of invasion and metastasis is observed in murine xenograft experiments [118] (Table 2).

#### 4.1.3. Semaphorin 3F

First observations that Semaphorin 3F (Sema3F) might have a role in cell motility and cell invasion was suggested by Brambilla and colleagues, in lung cancer cells [119]. Then, some studies reported that Sema3F can even induce apoptosis in cancer cells as well as tumor suppression in various xenograft experiments. Indeed, transfection of Sema3F in the murine fibrosarcoma cell line A9 and in HEY ovarian cell line suppresses tumor formation in nude mice, whereas no effect was observed after transfection of Sema3F in the small cell lung cancer cell line GLC45 [120]. When nude rats were orthotopically implanted with lung cancer cells transfected or not with Sema3F gene, all animals injected with cells expressing sema3f survived to 100 days whereas all the other rats died [121] (Table 2).

A role of Sema3F in tumor angiogenesis was then suggested. Implantation of BHK-21 (Baby Hamster Kidney-21) cells transfected with Sema3F concomitantly with cells producing VEGF-165 inhibited tumor-related angiogenesis in mice whereas no effect on angiogenesis was observed when BHK-21 cells transfected with empty vector were implanted with the same VEGF-165 producing cells [122]. Moreover, Sema3F transfection in the renal cell line HEK293 induced smaller tumors and a poorly-vascularized phenotype in xenograft experiments [122]. As a consequence, Sema3F and VEGF were rapidly considered to generate opposite activities. In fact, in highly metastatic melanoma cells, Sema3F completely inhibits metastasis *in vivo* and decreased the number of intra-tumor vessels, suggesting that Sema3F has huge potential in anti-angiogenic and anti-metastasis therapies [123] (Table 2). In addition, Sema3F can represent a powerful inhibitor of melanoma cell proliferation through its relation with NRP receptors [124].

Moreover, Sema3F blocks cell attachment and spreading in MCF7 and C100 breast cell lines, this effect depending on its interactions either with NRP1 or NRP2 [125] (Table 2).

#### 4.1.4. Semaphorin 3E

Although most class 3 semaphorins are considered to be TSG, it appears that others support opposite activities. Indeed, Semaphorin 3E (Sema3E) is described as an enhancer of tumor growth and metastasis *in vitro* and *in vivo* in xenograft experiments using breast cancer cells [126] (Table 2).

### 4.2. Cooperation with Growth Factor Receptors

#### 4.2.1. VEGFRs

**Figure 4.**
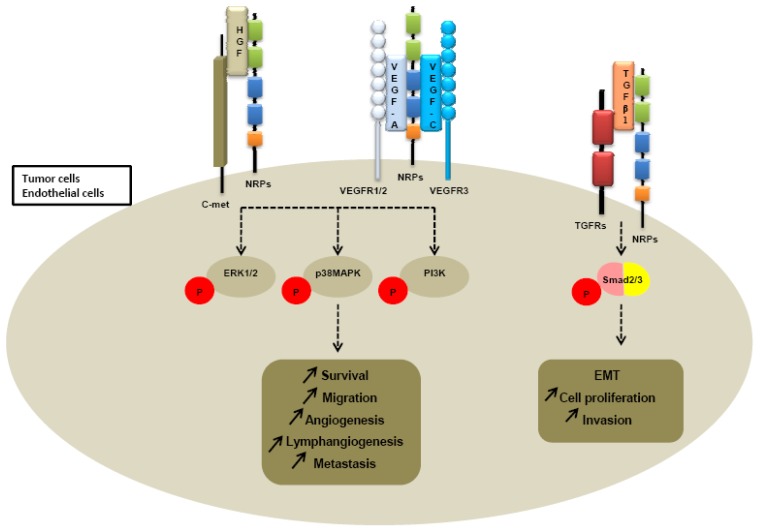
NRPs interactions with growth factor receptors.

Further investigations of neuropilin-dependent molecular pathways suggested that neuropilins contribute to tumor growth and angiogenesis through their cooperation with both VEGFR receptors, VEGFR1 and VEGFR2 (Figure 4).

First, Soker *et al.* reported that coexpression of NRP1 and VEGFR2 on porcine aortic endothelial cells enhances at least four-times the VEGF binding to VEGFR2 and in this way modulates downstream signaling and biological responses [21]. Later, Biacore analysis revealed that NRP1 interacts with both VEGFR1 and VEGFR2 [19]. Moreover, NRP1 enhances binding of VEGF to these two high affinity receptors. Similar results were obtained for NRP2. Indeed, co-immunoprecipitation studies revealed that NRP2 and VEGFR1 associate with each other to tranduce intracellular signals [129]. NRP2 enhances VEGFR1 phosphorylation and subsequently activates multiple intracellular pathways like extracellular signal-regulated kinase (ERK) or phosphatidylinositol 3-kinase (PI3K) pathways in colorectal cancer cells and pancreatic adenocarcinoma cells [45,46]. (Table 3) While NRP1 implication in the angiogenesis process has now considerable evidence, NRP2 appears to regulate lymphangiogenesis and metastatic processes. Indeed, NRP2 homozygous mutant mice are characterized by abnormal lymphatic and capillaries development proposing a selective requirement for NRP2 in the formation of lymphatic vessels [15]. Karpänen *et al.* propose that NRP2 contributes to lymphangiogenesis and metastastatic processes through direct interactions with VEGF-C, VEGF-D and VEGFR3 [20]. NRP2 increases VEGF-A and VEGF-C-induced survival and migration of endothelial cells [130]. Moreover, Caunt *et al.* recently reported that NRP2 blocking with a monoclonal antibody (anti-NRP2^B^) leads to a reduction of VEGFC-mediated migration of Lymphatic Endothelial cells (LEC) *in vitro* and to an inhibition of lymphangiogenesis *in vivo* [131]. Metastasis formation is found to be subsequently reduced in mice in xenograft models after anti-NRP2^B^ treatment [131]. Double-heterozygous *nrp2*^+^*^/^*^−^*vegfr2*^+^*^/^*^−^ mice have normal lymphatic development unlike double-heterozygous *nrp2*^+^*^/^*^−^*vegfr3*^+^*^/^*^−^ mice, indicating that Nrp2 partners with VEGFR3 to modulate lymphatic vessel sprouting and lymphangiogenesis [132]. Finally, another recently published study has reinforced the essential role of NRP2 in lymphangiogenesis process. Indeed, NRP2 knockdown by RNA interference improves corneal graft survival by suppressing lymphangiogenesis in vascular beds in a murine model of corneal transplantation [133] (Table 3).

#### 4.2.2. Integrins

Integrins have important roles in cell attachment, survival, migration, invasion and angiogenesis, which are all critical for carcinogenesis. Many integrins have been implicated in cancer progression. Indeed, Fukasawa and colleagues show that NRP1 interacts with integrin-β1 in pancreatic ductal adenocarcinoma and in this way promotes tumor cell growth, survival and invasion [134]. NRP1 was suggested to interact with α5β1 integrin to regulate angiogenesis in endothelial cells [135]. In lung cancer cells, anti-tumor effect of Sema3F is associated with loss of activated α5β3 integrin [121]. However, some integrins can support opposite activities. For example, in breast tumor cells, Sema3A treatment reduces cell migration in increasing α2β1 integrin level [127]. In endothelial cells, β3 integrin inhibits VEGF-mediated angiogenesis by sequestering NRP1 and preventing it from interacting with VEGFR2 [136].

#### 4.2.3. c-met

Because heparin growth factors FGF and HGF have been recently identified as NRPs ligands, they are believed to contribute to NRP-mediated angiogenesis too. Indeed, NRP1 potentiates HGF and FGF2 induced proliferation, survival, invasion in human umbilical vein endothelial cells (HUVEC), glioma cells, pancreatic cancer cells [23,137,138]. It appears that NRPs can be a receptor for HGF but can also enhance c-met phosphorylation by activating the c-met receptor itself. Indeed, co-immunoprecipitation studies confirm that NRPs interact directly with c-met receptor [138] (Table 3). Sulpice *et al.* confirmed in 2008 that both NRPs participate to VEGF and HGF linked-angiogenic activity in endothelial cells through enhancing autocrine hepatocyte growth factor (HGF)/scatter factor (SF)/c-Met signaling [24,137]. NRPs generate activation of several signaling pathways through c-met interaction, including p38-mitogen-activated protein kinase (p38-MAPK), extracellular signal-regulated kinase (ERK), src, phosphatidylinositol 3-kinase (PI3K) [24,137,138] (Figure 4).

#### 4.2.4. TGFRs

More recently, a study suggested that NRP1 is a receptor for both active TGFβ1 and TGFβ1-LAP. In addition, NRP1-TGFβ1 interactions on T cells resulted in enhanced T regulator activity [139]. Then other reports confirmed that NRP1 promotes TGFβ1 signaling pathway. Indeed, in a recent study, Glinka *et al.* show that NRP1 associates with TGFRI and TGFRII to enhance TGFβ1 signaling in cancer cells [140] (Table 3). Moreover, NRP1 was shown to confer a myofibroblast phenotype by enhancing PDGF/TGFβ1 pathways in hepatic human cells [141] and in stromal fibroblasts [142]. Because NRPs are not tyrosine-kinase receptors, NRP1 was thought to cooperate with TGFRs to transduce the signal [142]. A similar role was attributed to NRP2. Indeed, we noticed that NRP2 expression enhances TGFβ1 signaling leading to constitutive Smad2/3 phosphorylation in colorectal cancer cells [143]. Biacore analysis revealed that NRP2, like NRP1, is a receptor for active TGFβ1 [143]. Moreover, NRP2 confered a fibroblastic-like shape to cancer cells, suggesting an involvement of neuropilin-2 in epithelial mesenchymal transition (EMT) [143] (Table 3). EMT is indeed characterized by a breakdown of cell junctions and the loss of epithelial characteristics and cell polarity, contributing to carcinoma progression. Besides the gain of mesenchymal markers, EMT endows cancer cells for migration, invasiveness and subsequent metastasis formation [144]. Indeed, the presence of neuropilin-2 in colorectal carcinoma cell lines is correlated with loss of epithelial markers such as cytokeratin-20 and E-cadherin and with acquisition of mesenchymal molecules such as vimentin [143].

In view of its implication in multiple processes such as angiogenesis, lymphangiogenesis, EMT, and metastasis, NRP2 fulfills all the criteria of a therapeutic target to disrupt multiple oncogenic functions in solid tumors.

## 5. Neuropilins: A Surrogate Marker for Cancer Progression

Because NRP2 is implicated in multiple processes including angiogenesis, lymphangiogenesis and metastasis, it became rapidly apparent that NRP2 detection constitutes a novel diagnostic and prognostic tool in a great majority of tumors.

NRP2 expression is correlated twith increased vascularity and poor prognosis in osteosarcomas [39] and non small cell lung carcinoma (NSCLC) [41]. Nrp2 was also detected in salivary adenoid cystic carcinomas (SACCs), and its expression level significantly correlated with microvessel density, tumor size, clinical stage, vascular invasion, and metastasis of SACCs [54]. In breast cancers, NRP2 expression is significantly correlated with lymph node metastasis, VEGF-C expression and cytoplasmic CXCR4 expression [50]. NRP2 expression is significantly upregulated in early and advanced stages of neuroblastomas [44]. Moreover, NRP2 is expressed by a vast majority of endocrines pancreatic tumors, suggesting that NRP2 can be used as a diagnostic marker for these tumors [47]. NRP2 was shown to be also a biomarker of potential clinical significance associated with bladder cancer progression [55].

## 6. Neuropilins Targeting

**Figure 5.**
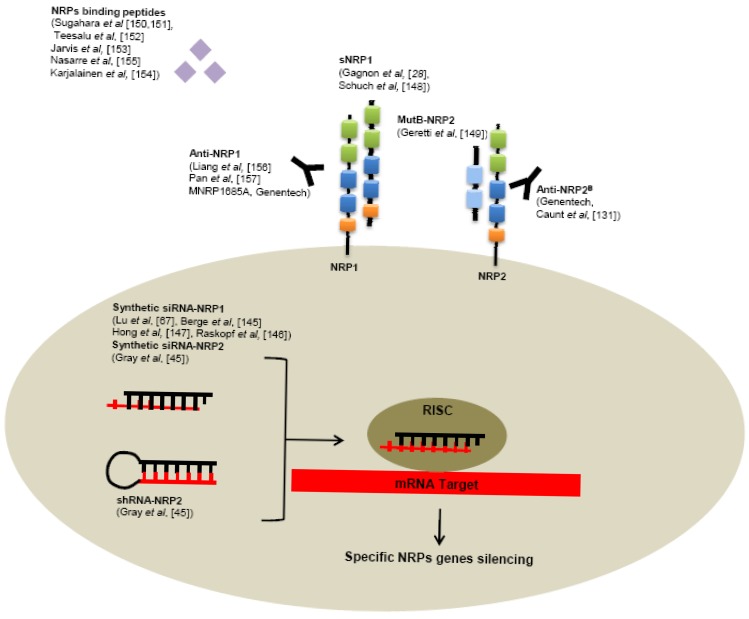
Biotechnological tools developped to target NRPs. Preclinical studies demonstrated the potential interest of several strategies to inhibit oncogenic functions induced by NRPs including: small interfering RNA, peptides, soluble NRPs antagonists, monoclonal antibodies (RISC: «RNA-Induced Silencing Complex»).

Several tools have been developed to neutralize NRPs receptors, targeting NRPs genes like RNA interference or receptors using specific monoclonal antibodies or small peptides (Figure 5).

### 6.1. RNA Interference

Use of siRNA targeting NRP1 significantly reduces tumor growth, angiogenesis, metastasis formation in various human cancer models, such as hepatocellular carcinoma [145,146], acute myeloid leukemia [67], lung cancer [147]. Also reduction of NRP2 expression by shRNA in colorectal cancer cells induces smaller tumors, decreased number of metastases and enhanced apoptosis in comparison with control shRNA in a murine xenograft model [45]. In addition, intraperitoneally treatment of tumor bearing mice with liposomes containing NRP2 siRNA reduces tumor growth and metastasis [45].

### 6.2. Small Molecules

As seen previously, alternative splicing generates naturally occurring soluble forms sNRP1 and sNRP2. These soluble sNRP are first described as inhibitory molecules, functioning as natural ligand trap, inhibiting their interaction with membrane receptors. Soluble neuropilins lack the transmembrane segment and intracellular domain. Gagnon *et al.* reported that overexpression of sNRP1 in Dunning rat prostate carcinoma cell lines AT2.1 and AT3.1 generates tumors with large and hemorrhagic center, with decreased proliferation and increased apoptosis in rats [28]. Moreover, sNRP1 inhibits the binding of VEGF_165_ to full-length NRP1 [28].

Schuch *et al.* confirmed these findings in a murine sarcoma model using NMuMG/VEGF and NMuMG/sNRP-1 cells that have been engineered to produce high levels of recombinant VEGF and sNRP1 [148]. VEGF treatment resulted in tumor growth and vascularization, whereas treatment with soluble NRP-1 (sNRP-1) inhibited tumor angiogenesis and growth. Moreover, in a systemic leukemia model, survival of mice injected with adenovirus (Ad) encoding for Fc-sNRP-1 (sNRP-1 dimer) was significantly prolonged as compared with control mice [148].

Since naturally occurring soluble forms of neuropilins are described to inhibit tumor progression, researchers tend nowadays to develop soluble peptides preventing VEGF-binding on neuropilins. For this purpose, Geretti *et al.* described very recently a mutant of the B-domain of NRP2 (MutB-NRP2) with 8-fold increased affinity for VEGF compared to wild-type B domain of NRP2 [149]. This MutB-NRP2 significantly inhibits tumor growth in a xenograft model using melanoma cells, alone and in combination with bevacizumab [149].

Furthermore, screening of phage libraries expressing random peptides binding to various cancer cells has allowed the identification of amino acid sequences especially binding NRPs. Indeed, Sugahara and collaborators reported two tissue-penetrating peptides binding human integrins and NRP1 capable of penetrating into tumor tissue and cells [150,151]. Conjugation of these peptides to anti-tumor drugs or imaging agents might enhance tumor imaging and the activity of anti-tumor therapies [150-152]. Since then, another peptide targeting NRP1 has been described in various model of cancers cell *in vitro* [153-155].

### 6.3. Monoclonal Antibodies

Genentech has very recently developed monoclonal antibodies targeting NRP1. In particularly, high-affinity monoclonal antibodies targeting either CUB domains (anti-NRP1^A^) or coagulation factors V/VIII domains (anti-NRP1^B^) of NRP1 have been first generated. [156] These anti-NRP1 antibodies induce reduction of VEGF-induced migration of HUVEC cells and inhibit tumor formation in animal models [156]. Later, anti-NRP1 monoclonal antibodies were shown to block VEGF-binding to NRP1 and to have an additive effect with anti-VEGF therapies to reduce tumor growth [157].

One of them, a full human antibody targeting NRP1, MNRP1685A is actually in phase-1 of development alone or in combination with bevacizumab with or without paclitaxel for treatment of advanced solid tumors [158].

Monoclonal antibodies targeting the b1/b2 domains of NRP2 have been recently developed. By blocking binding of VEGF and VEGFC to NRP2, these anti-NRP2^B^ monoclonal antibodies decrease the number of tumor-associated lymphatic vessels and metastasis in sentinel lymph node and in distant organs in mice xenograft experiments [131].

### 6.4. Semaphorins

NRPs role in tumorigenesis is more complex than initially thought and appears to depend on the nature of the ligand. In the context of cancer, it appears that semaphorins and VEGF are competing for NRPs binding, although they bind different NRPs sub-units. While semaphorins are responsible for inhibition of tumor growth, proliferation and even induction of apoptosis in cancer cells, VEGF tends to oppositely enhance angiogenesis and tumor growth. As described above, some semaphorins such as Sema3B and Sema3F are considered as TSG and are very often downregulated in cancer cells [102,104,120]. Overexpression of Sema3 genes may represent a promising new type of therapy for preventing tumor angiogenesis, growth, and metastasis. Moreover, other semaphorins such as Sema3E or Sema4D function as pro-angiogenic and pro-oncogenic molecules [89,126,159,160]. Neutralization of these molecules or their relative receptors thus may represent a new therapeutic strategy for cancer treatment. In particular, one monoclonal antibody VX15/2503 binding to the sema4D is currently in phase-1 of development for the treatment of advanced solid tumors [161]. Therapeutic use of semaphorin pathway seems to represent one of the major therapeutic strategies considered, capable of antagonizing VEGF-mediated angiogenesis and tumor progression [88].

## 7. Conclusions

NRPs are multifunctional non-tyrosine kinase receptors for class-3 semaphorins and VEGF family members implicated in both physiological development and pathological situations. NRPs are expressed in endothelial cells and in many types of cancer cells. Through their direct interactions with plexins or growth factor receptors, NRPs have rapidly emerged as key regulators of angiogenesis, lymphangiogenesis, EMT and tumor progression. In many cancers, expression of one or both has been correlated with tumor progression and/or poor prognosis. As a consequence, several strategies have been used in pre-clinical studies to inhibit NRPs function, such as knockdown strategies with siRNAs, small peptide inhibitors, and blocking antibodies. However, the molecular mechanisms by which NRPs modulate cancer progression are still poorly understood. Understanding the interactions between VEGF, VEGFRs, semaphorins and NRPs should provide additional data for the rational development of novel anti-tumor strategies.

## Figures and Tables

**Table 1.** Neuropilins (NRPs) expression in cancer cells.

**Tumors**		**NRP1**		**NRP2**		**References**	
**Brain tumors**							
**Astrocytomas**		x		ND		Ding H *et al.*, 2000 [56]	
**Neuroblastomas**		x		x		Fakhari M *et al.* [44]	
**Gliomas**		x		x		Rieger J *et al.*, 2003 [43]	
		x		ND		Osada H *et al.*, 2004 [54]	
**Glioblastomas**		x		ND		Broholm H *et al.*, 2004 [57]	
**Pituitary tumors**		x		ND		Onofri C *et al.*, 2006 [58]	
**Digestive tumors**							
**Endocrine pancreatic tumors**		ND		x		Cohen T *et al.*, 2002 [47]	
**Pancreatic adenocarcinomas**		x		ND		Parikh AA *et al.*, 2003 [59]	
		x		x		Fukahi K *et al.*, 2004 [48]	
		x		x		Li M *et al.*, 2004 [49]	
		x		ND		Feurino LW *et al.*, 2007 [60]	
		x		x		Dallas NA *et al.*, 2008 [46]	
**Gastric cancer**		x		ND		Akagi M *et al.*, 2003 [61]	
		x		ND		Hansel DE *et al.*, 2004 [62]	
**Colon cancer**		x		ND		Parikh AA *et al.*, 2004 [63]	
		x		ND		Ochiumi T *et al.*, 2006 [64]	
		ND		x		Gray MJ *et al.*, 2008 [45]	
**Leukemias**							
**Acute Myeloid Leukemia (AML)**		x		ND		Kreuter M *et al.*, 2006 [65]	
		x		ND		Kreuter M *et al.*, 2007 [66]	
		x		x		Vales A *et al.*, 2007 [51]	
		x		ND		Lu L *et al.*, 2007 [67]	
**Chronic lymphocytic leukemia B**		x		ND		Nowakowski GS *et al.*, 2008 [68]	
**Other solid tumors**							
**Breast cancers**		x		ND		Stephenson JM *et al.*, 2002 [69]	
		x		ND		Ghosh M *et al.*, 2008 [70]	
**NSCLC**		x		x		Kawakami T *et al.*, 2002 [41]	
		x		x		Lantuejoul S *et al.*, 2003 [71]	
**Lung cancers**		x		x		Tomizawa *et al.*, 2001 [42]	
**Melanomas**		x		x		Lacal PM *et al.*, 2000 [40]	
		x		ND		Straume O *et al.*, 2003 [72]	
**Prostate cancers**		x		ND		Latil A *et al.*, 2000 [73]	
		x		ND		Vanveldhuizen PJ *et al.*, 2003 [74]	
**Laryngeal carcinomas and papillomas**		x		ND		Zhang S *et al.*, 2006 [75]	
**Salivary adenoid cystic carcinoma**		ND		x		Cai Y *et al.*, 2010 [52]	
**Infantile hemangiomas**		ND		x		Calicchio ML *et al.*, 2009 [53]	
**Ovarian carcinomas**		x		ND		Hall GH *et al.*, 2005 [76]	
		x		x		Osada R *et al.*, 2006 [54]	
		x		ND		Baba T *et al.*, 2007 [77]	
**Bladder cancers**		ND		x		Sanchez Carbayo M *et al.*, 2003 [55]	
**Osteosarcomas**		ND		x		Handa *et al.*, 2000 [39]	

**Table 2.** Class 3 semaphorins expression and function in tumor cells.

**Semaphorins**	**Cells**	**Activity**	**References**
**Sema3A**	Neural progenitor cells	Induction of cell repulsion and cell death	Bagnard D, 2001[109]
	Endothelial cells	Induction of apoptosis	Guttmann-Raviv N, 2007 [110]
	Leukemic T cells	Relocalization of Fas into the lipid raft	Moretti S, 2008 [111]
	Breast cancer cells	Inhibition of tumor growth, of intra-tumor vasculature	Kigel B, 2008 [112]
	Breast tumor cells	Inhibition of cell migration, increase of alpha2beta1 integrin level	Pan H, 2009 [127]
**Sema3B**	murine pancreatic cells	Inhibition of tumor growth, of intra-tumor vasculature	Maione F, 2009 [113]
	murine mammary carcinoma cells	Inhibition of tumor growth, of intra-tumor vasculature and metastasis	Casazza A, 2011 [114]
	Lung cancer cells	Inhibition of growth and induction of apoptosis	Tomizawa, 2001 [42]
	Ovarian adenocarcinoma cell line	Diminution of tumorigenicity in xenografts experiments, diminution of colony formation and cell proliferation	Tse C, 2002 [115]
	Lung and breast cancer cells	Induction of apoptosis	Castro-Rivera E, 2004, 2008 [116, 117]
	Breast cancer cells	NRP1-sema3B interactions increase IL8 production in tumor cells, promoting invasion and metastasis	Rolny C, 2008 [118]
**Sema3D**	Breast cancer cells	Inhibition of tumor progression	Kigel B, 2008 [112]
**Sema3E**	Breast cancer cells	Increase of tumor growth, metastasis	Christensen C, 2005 [126]
**Sema3F**	Lung cancer cells	Role in cell motility and cell adhesion	Brambilla E, 2000 [119]
	Small cell lung cancer cells, ovarian adenocarcinoma	Diminution of tumorigenicity in xenografts experiments, induction of apoptosis	Xiang R, 2002 [120]
	Breast cancer cells	Inhibition of cell migration	Nasarre P, 2003 [128]
	Endothelial, renal cancer cells	Inhibition of cell proliferation, inhibition of angiogenesis *in vivo*	Kessler O, 2004 [122]
	Melanomas	Inhibition of metastasis, of intra-tumor vessels and induction of large areas of apoptosis *in vivo*	Bielenberg BR, 2004[123]
	Breast cancer cells	Induction of cell repulsion, inhibition of cell contacts and proliferation	Nasarre P, 2005 [125]
	Lung cancer cells	Enhances survival in xenografts experiment	Kusy S, 2005 [121]
	Melanomas	Inhibition of cell proliferation	Chabbert-de Ponnat I, 2006 [124]
	Breast and melanoma cancer cells	Inhibition of tumor progression *in vivo*	Kigel B, 2008 [112]

**Table 3.** NRPs interactions with growth factor receptors.

**Complexes**	**Cells**	**Activity**	**References**
**NRP/VEGFR1**	Biacore analysis	NRP1 associates with VEGFR1 and VEGFR2	Fuh *et al.*, 2000 [19]
	Endothelial Porcine Aortic Endothelial (PAE) cells	NRP2 co-immunoprecipitates with VEGFR1	Gluzman-Poltorak *et al.*, 2001 [129]
	Colorectal cancer cells	NRP2 enhances VEGFR1 phosphorylation, migration, invasion in tumor cells through PI3K and ERK activation. Targeting NRP2 with shRNA reduces tumor growth, metastasis formation in xenograft experiments.	Gray *et al.*, 2008 [45]
**NRP/VEGFR2**	Pancreatic Adenocarcinoma cancer cells	NRP2 enhances VEGFR1 phosphorylation, migration, invasion in tumor cells through PI3K and ERK activation. Reduced NRP-2 expression decreases migration, invasion, and anchorage-independent growth. Targeting NRP2 with shRNA reduces tumor growth, tumor vasculature and metastasis formation in xenograft experiments.	Dallas *et al.*, 2008 [46]
	Endothelial Porcine Aortic Endothelial (PAE) cells	NRP1 enhances the binding of VEGF to VEGFR2	Soker *et al.*, 1998 [21]
	Biacore analysis	NRP1 associates with VEGFR1 and VEGFR2	Fuh *et al.*, 2000 [19]
	293T, PAE, human microvascular endothelial cells	NRP2 interacts with VEGFR2 and VEGFR3 and enhances their activation. NRP2 overexpression enhances VEGF-A and VEGF-C induced survival and migration of human endothelial cells.	Favier *et al.*, 2006 [130]
	Lymphatic endothelial cells	NRP2 interacts with VEGFR2 and VEGFR3, enhances their phosphorylation and activation.	Caunt *et al.*, 2008 [131]
**NRP/VEGFR3**	Lymphatic endothelial cells and transfected 293T	NRP2 interacts with VEGFR3 in co-immunoprecipitation studies.	Karpänen *et al.*, 2006 [20]
	293T, PAE, human microvascular endothelial cells	NRP2 interacts with VEGFR2 and VEGFR3 and enhances their activation. NRP2 overexpression enhances VEGF-A and VEGF-C induced survival and migration of human endothelial cells.	Favier *et al.*, 2006 [130]
	Lymphatic endothelial cells	NRP2 interacts with VEGFR2 and VEGFR3, enhances their phosphorylation and activation.	Caunt *et al.*, 2008 [131]
**NRP/c-met**	HUVEC	HGF binds NRP1 and NRP2. NRP1 and NRP2 enhance c-met phosphorylation and migration through ERK activation.	Sulpice *et al.*, 2008 [24]
	Glioma	NRP1 promotes glioma progression through activation of HGF/SF autocrine pathway and ERK pathway activation.	Hu B *et al.*, 2007 [137]
	Pancreatic cancer cells	NRP1 interacts with c-met, promoting invasion through ERK and p38MAPK activation.	Matsushita *et al.*, 2007 [138]
**NRP/TGFR**	Stromal fibroblasts	NRP1 enhances Smad activation and induces a myofibroblast phenotype.	Cao *et al.*, 2010 [142]
	Breast cancer cells	NRP1 and NRP2 associate with TGFRI and TGFRII and enhance Smad2/3 phosphorylation.	Glinka *et al.*, 2010 [140]
	Colorectal cancer cells	NRP2 interacts with TGFRI and enhances Smad2/3 activation. NRP2 induces a TGFβi-dependant Epithelial Mesenchymal Transition in colorectal cancer cells.	Grandclement *et al.*, 2010 [143]
